# Assessing genomic diversity and signatures of selection in Pinan cattle using whole-genome sequencing data

**DOI:** 10.1186/s12864-022-08645-y

**Published:** 2022-06-21

**Authors:** Shunjin Zhang, Zhi Yao, Xinmiao Li, Zijing Zhang, Xian Liu, Peng Yang, Ningbo Chen, Xiaoting Xia, Shijie Lyu, Qiaoting Shi, Eryao Wang, Baorui Ru, Yu Jiang, Chuzhao Lei, Hong Chen, Yongzhen Huang

**Affiliations:** 1 Key Laboratory of Animal Genetics, Breeding and Reproduction of Shaanxi Province, College of Animal Science and Technology, Northwest A&F University, No. 22 Xinong Road, Yangling Shaanxi, 712100 China; 2 Institute of Animal Husbandry and Veterinary Science, Henan Academy of Agricultural Sciences, Zhengzhou Henan, 450002 China; 3 Henan Provincial Animal Husbandry General Station, Zhengzhou Henan, 450008 China

**Keywords:** Pinan cattle, WGS, Genetic diversity, Population Structure, DCMS

## Abstract

**Background:**

Crossbreeding is an important way to improve production beef cattle performance. Pinan cattle is a new hybrid cattle obtained from crossing Piedmontese bulls with Nanyang cows. After more than 30 years of cross-breeding, Pinan cattle show a variety of excellent characteristics, including fast growth, early onset of puberty, and good meat quality. In this study, we analyzed the genetic diversity, population structure, and genomic region under the selection of Pinan cattle based on whole-genome sequencing data of 30 Pinan cattle and 169 published cattle genomic data worldwide.

**Results:**

Estimating ancestry composition analysis showed that the composition proportions for our Pinan cattle were mainly Piedmontese and a small amount of Nanyang cattle. The analyses of nucleotide diversity and linkage disequilibrium decay indicated that the genomic diversity of Pinan cattle was higher than that of European cattle and lower than that of Chinese indigenous cattle. De-correlated composite of multiple selection signals, which combines four different statistics including θπ, CLR, F_ST_, and XP-EHH, was computed to detect the signatures of selection in the Pinan cattle genome. A total of 83 genes were identified, affecting many economically important traits. Functional annotation revealed that these selected genes were related to immune (*BOLA-DQA2*, *BOLA-DQB*, *LSM14A*, *SEC13*, and *NAALADL2*), growth traits (*CYP4A11*, *RPL26*, and *MYH10*), embryo development (*REV3L*, *NT5E*, *CDX2*, *KDM6B*, and *ADAMTS9*), hornless traits (*C1H21orf62*), and climate adaptation (*ANTXR2*).

**Conclusion:**

In this paper, we elucidated the genomic characteristics, ancestry composition, and selective signals related to important economic traits in Pinan cattle. These results will provide the basis for further genetic improvement of Pinan cattle and reference for other hybrid cattle related studies.

**Supplementary Information:**

The online version contains supplementary material available at 10.1186/s12864-022-08645-y.

## Background

Cattle are one of the most important domestic animals for human beings. According to the origin of cattle, domestic cattle can be simply divided into two subspecies: *Bos taurus* and *Bos indicus* [1]*.* Or according to geographical region, it can also be divided into five groups: European taurine, Eurasian taurine, East Asian taurine, Chinese indicine, and Indian indicine [2]. The earliest domesticated cattle were mainly used for draft purposes. With the development of society, except for a few developing countries, cattle have been developed in the direction of specialization through continuous breeding and improvement. Today, there are hundreds of cattle breeds in the world, and China alone has more than 50 native breeds, including five excellent breeds (Yanbian, Luxi, Qinchuan, Jinnan, and Nanyang cattle). Because the cattle in China used to be mainly used for farming, the production performance is not as good as the specialized beef cattle breeds in European and American countries. To compensate for the perceived lower beef production potential of local cattle, these cattle are often crossed with exotic breeds to develop new breeds. Piedmontese cattle, which are native to Italy, are internationally recognized as terminal male due to their double muscle genes and have been introduced to more than 20 countries for crossbreeding. Grading up is often used to improve the performance of cattle. A new breed called Pinan cattle has been developed in Henan Province of China by introducing Piedmontese cattle as male parents and grading up with local Nanyang cattle. It is generally used for production after crossing three generations. Pinan cattle has many advantages, such as the early onset of puberty, fast-growth speed, and good meat quality, which greatly improved the breeding efficiency and meet the demands of people.

Whole-genome sequencing (WGS) has been used to find a large number of variations that can be used as molecular genetic markers. The application of WGS plays an important role in the study of human diseases, the breeding of animals and plants, as well as the origin and domestication of species. WGS studies have yielded many important results in the field of livestock, including domestication, adaptive mechanisms of indigenous breeds, candidate genes responsible for important economic traits, and historical population dynamics [3, 4]. In earlier years, it was mainly European commercial cattle that were studied using whole-genome sequencing [5]. Later, researchers also began to look at native varieties to find genomic variations associated with desired traits in native varieties [6–10], which could help improve breeding.

In the current study, we sequenced the genomes of 30 Pinan cattle to detect genetic variation, population structure, and selective sweep. Our result will lay a foundation for further studies on the genetic basis of important economic traits, provide ideas and basis for future improvement in Pinan cattle, and provide a reference for related studies in other hybrid breeds.

## Results

### Sequencing and SNP detection

A total of 5,402,336,411 clean reads were generated after genome sequencing in 30 Pinan cattle samples. The clean reads were aligned to the latest *Bos taurus* reference genome (ARS-UCD1.2) with an average depth of 9.67X using Sentieon [11] software. We identified 23,077,494 biallelic SNPs in 30 Pinan cattle using GATK. Among these SNPs, functional annotation showed that SNPs mainly existed in the intergenic region (59.6%) and intronic region (37.4%). Exons accounted for 0.77% of the total SNPs, including 71,193 non-synonymous SNPs and 99,811 synonymous SNPs (Table S3).

To place Pinan cattle into a global context, we also detected the single nucleotide polymorphisms in five "core" cattle populations [2], and two Chinese cattle breeds (Qinchuan and Jiaxian Red cattle). Among these breeds, Chinese indicine (37,087,338) has the highest number of SNPs, followed by crossbreed Jiaxian cattle (29,315,624), Qinchuan cattle (26,270,077), Nanyang cattle (25,468,742), Pinan cattle (23,077,494) and Indian indicine (23,005,288). The number of SNP in Bos taurus was lower than that in indicine cattle and hybrid cattle. The results of this SNP distribution are consistent with the observations of other studies [2, 8].

### Population structure analysis

**Fig. 1:**
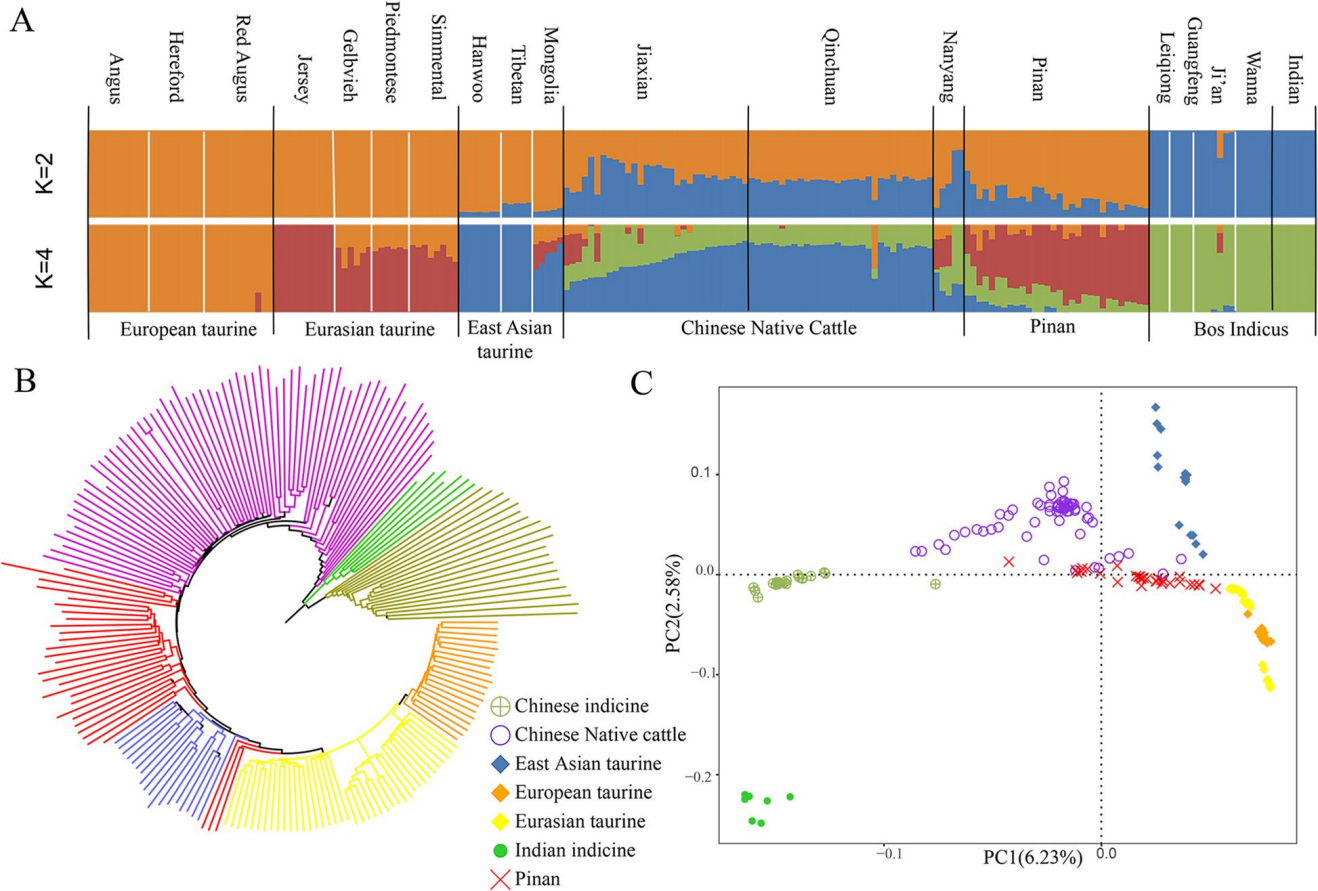
Population structure of Pinan cattle and its relationship with several breeds in the world. (A) ADMIXTURE was used with K = 2 and K = 4 for model-based clustering among different cattle. Color them by geographical area and label them with the breed. Neighbor-joining trees (B) and principal component analysis (C) separated the cattle breeds (199 animals in total) into seven categories

To explore the genetic relationships between Pinan cattle and other cattle breeds around the world, estimating ancestry, building neighbor-joining (NJ) trees and principal component analysis (PCA) were performed (Fig. 1). The first and second PCs explained 6.23% and 2.58% of the variation in the entire genomic data, respectively. The first PC separated *Bos taurus* and *Bos indicus.* The analysis revealed clear geographical patterns of cattle distribution. The proportions of individuals in Pinan cattle inferred by the ADMIXTURE are presented in Fig. 1 A. The case with a small CV error value (Table S4) was selected for drawing. When K = 2, these different cattle breeds can be divided into two groups: *Bos taurus* and *Bos indicus*; when K = 4, European taurine and Zebu cattle have a single genomic composition. We can see that the ancestry proportions inherited from *Bos taurus* are greater than that from *Bos indicus* in Pinan cattle. To be more precise, Pinan cattle composition is more similar to Eurasian taurine which indicates that the genetic influence of Piedmontese cattle was greater than that of Nanyang cattle. The results of the NJ tree (Fig. 1B and Figure S1) and PCA (Fig. 1C) analysis were similar. All "core" cattle groups form independent clusters. The individuals of the Pinan cattle are also mostly clustered together and close to the Piedmontese cattle.

### Genetic diversity and linkage disequilibrium

**Fig. 2:**
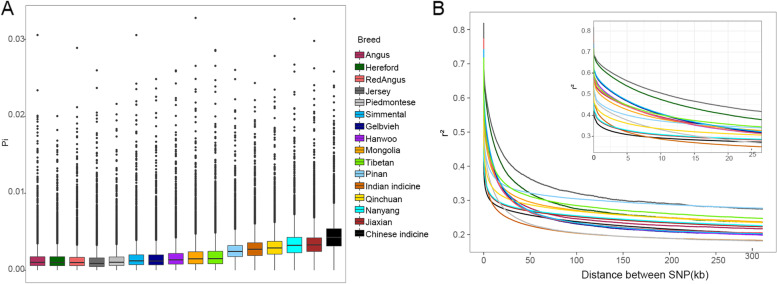
A The nucleotide diversity of 12 different cattle breeds. The black line in the boxplot is the median line and the outside points are outliers. B Genome-wide average LD decay is estimated from each breed. Different colored lines represent different breeds. The legend in the middle is shared by both figures

To elucidate the genomic characteristics in Pinan cattle, nucleotide diversity was calculated (Fig. 2A). The results showed that Chinese indicine had the highest nucleotide diversity, followed by Nanyang, Indian, and Qinchuan cattle. The lowest nucleotide diversity was found in European cattle. The nucleotide diversity of our crossbreed Pinan cattle is lower than that of female parent Nanyang cattle but higher than that of male parent Piedmontese cattle. From the perspective of linkage disequilibrium (LD) (Fig. 2B), although the LD of each variety decreased rapidly, there were some differences among varieties. At distances between markers (> 50 kb), Indian indicine and Chinese indicine had the lowest LD level. The highest LD level in Jersey, followed by Pinan. East Asian taurine and European taurine had a medium LD level. Overall, the LD level of indicine breeds was lower than that of taurine breeds, consistent with previous studies [12]. It is worth noting that Pinan cattle had a higher LD level than either of its parents.

### De-correlated composite of multiple signals

**Fig. 3:**
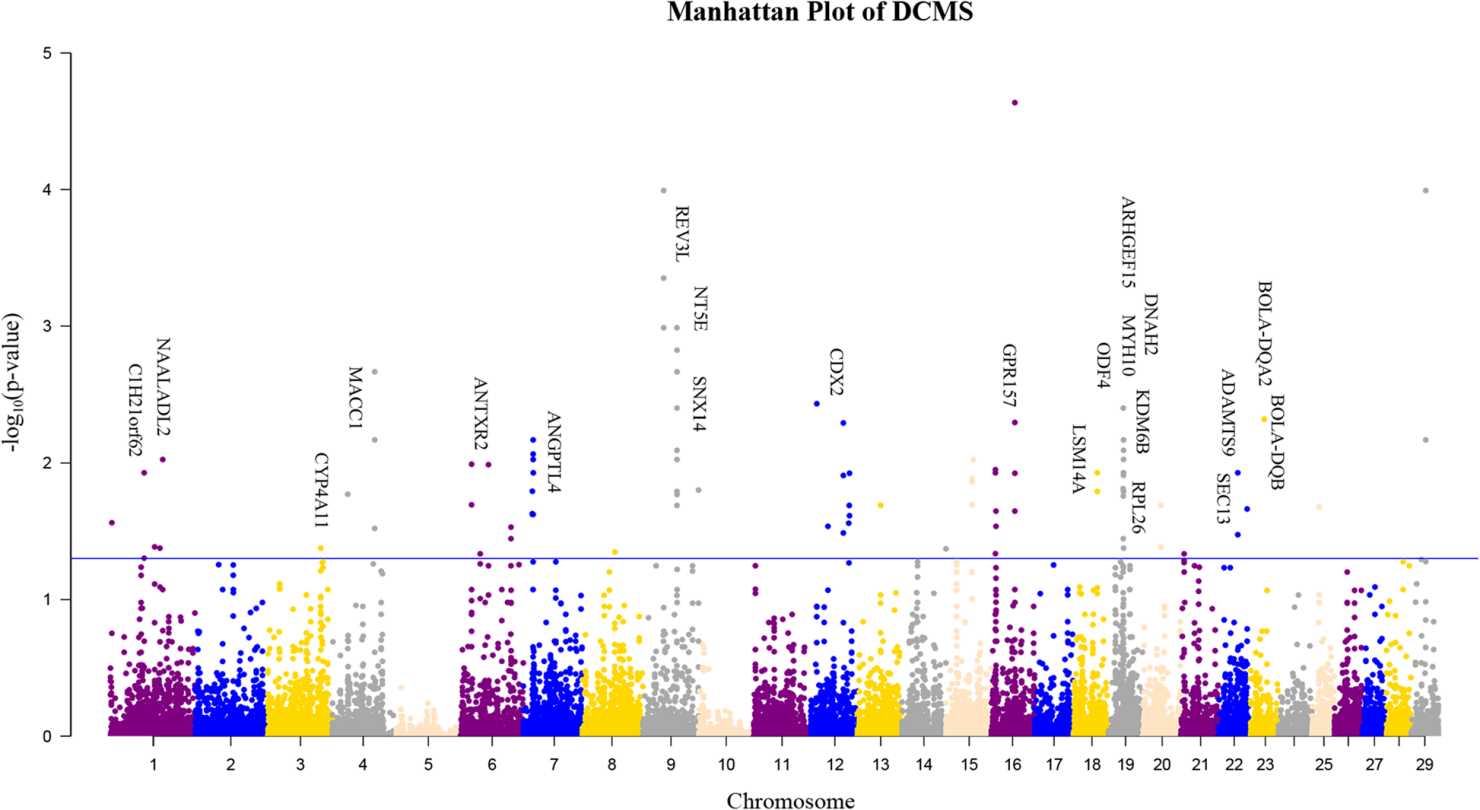
Manhattan plot of the selective signals detected by the DCMS method in the Pinan cattle. The dashed lines indicade the significant threshold level at a FDR of 5% (q-value < 0.05)

Within-population and cross-population statistics were combined separately in a single score using the De-correlated composite of multiple signals (DCMS) statistic [13]. After calculation of the DCMS statistics, the p-value was fitted to a normal distribution and corrected for multiple testing (FDR < 0.05). The analysis identified 92 candidate regions (Table S5) of the genome containing 83 genes. We identified several of the most significant genomic regions on BTA 9: 63.80–64.09 M and BTA 19:27.50 M-28.19 M (Fig. 3). All regions were annotated and some candidate genes associated with important traits were found (Table 1). Including hornless traits (*C1H21orf62*), growth traits (*CYP4A11*, *RPL26*, and *MYH10*), Disease-related traits (*MACC1* and *SNX14*), climate adaptation (*ANTXR2*), lipid metabolism (*ANGPTL4*), embryo development (*REV3L*, *NT5E*, *CDX2*, *KDM6B*, and *ADAMTS9*), immune response (*BOLA-DQA2*, *BOLA-DQB*, *LSM14A*, *SEC13*, and *NAALADL2*), sperm flagella (*DNAH2*), milk yield (*ODF4*, *ARHGEF15*, and *MYH10*).

**Table 1:** Genomic regions detected through the DCMS analyses in Pinan cattle

Region (Mb)	q-value	Candidate gene	Trait	Citation
1: 27.40–27.90	0.027351	*PAXBP1, C1H21orf62*	hornless	[14]
1: 82.84–82.89	0.041116	*FAM131A, EIF4G1, PSMD2, CLCN2*	disease-relate	[15]
1: 92.80–92.85	0.042031	*NAALADL2*	embryo development	[16]
3: 00.18–99.23	0.042031	*CYP4A11*	growth traits	[17]
4: 28.90–28.95	0.016919	*MACC1*	colorectal carcinoma	[18]
6: 37.14–37.19	0.046225	*MED28, LAP3, FAM184B*	disease-relate, milk production	[19, 20]
6: 94.72–94.79	0.035810	*ANTXR2*	climate adaptation	[21]
7: 17.00–17.09	0.021124	*ANGPTL4, RAB11B, MARCH2, HNRNPM,*	lipid metabolism	[22]
7: 18.44–18.55	0.012144	*CAPS, VMAC, NDUFA11, RANBP3, FUT6, NRTN, PRR22*	disease-relate	[23]
7: 18.74–18.79	0.011793	*LOC112447361*		
8: 60.72–60.77	0.044877	*MELK*	disease-relate	[24]
9: 38.98–39.07	0.000525	*REV3L*	embryo development	[25]
9: 63.80–64.07	0.008056	*SYNCRIP, SNX14, NT5E*	disease-related, immune suppressor	[26–28]
9: 104.28–104.33	0.015762	*LOC100300342*		
12: 11.26–11.31	0.003701	*WBP4*	disease-related	[29]
12: 32.06–32.11	0.029085	*URAD, CDX2, FLT3*	embryo development, hematopoiesis	[30, 31]
12: 71.96–72.01	0.020448	*LOC100337076*		
12: 72.44–72.49	0.024298	*LOC112449072*		
13: 43.42–43.47	0.020407	*LOC112441538, LOC112449293*		
15: 48.48–48.57	0.015635	*LOC781483, LOC781444, LOC781403*		
16: 8.92–8.97	0.022505	*LOC789494*		
16: 44.32–44.41	0.008253	*GPR157, LOC107131287*	neuronal differentiation	[32]
18: 44.60–44.67	0.013972	*LSM14A*	antiviral immune response	[33]
19: 27.08–27.13	0.015391	*NLGN2, SPEM2, LOC112442746, TMEM102, TMEM256, SPEM1, FGF11, CHRNB1*	disease-related	[34–38]
19: 27.50–28.19	0.015021	*TMEM88, NAA38, CYB5D, DNAH2, KDM6B, RANGRF, SLC25A35, ARHGEF15, ODF4, KRBA2, RPL26, RNF222, NDEL1, MYH10*	disease-related, growth traits, sperm flagella, milk yield	[39–47]
21: 6.20–6.50	0.046249	*CERS3*	Congenital Ichthyosis	[48]
22: 36.84–36.91	0.022639	*ADAMTS9*	embryo development	[49]
22: 54.36–54.41	0.021701	*SEC13, ATP2B2*	immune response	[50, 51]
23: 25.58–25.63	0.004795	*BOLA-DQA2, BOLA-DQB*	immune response	[52]
25: 13.46–13.51	0.020943	*PARN*	disease-related	[53]
29: 27.34–27.39	0.006772	*LOC787625*		
29: 27.42–27.47	0.000102	*LOC787694*		

## Discussion

The characterization of population structure and genetic diversity can help us to evaluate bovine genetic resources and play an important role in utilization and conservation. In this study, the population genetic structure of Pinan Cattle was studied in the background with different cattle breeds. As can be seen from the ADMIXTURE analysis (Fig. 1), the ancestral contributions of Pinan cattle were mainly from Piedmontese.

Although Pinan cattle are closer to the Piedmontese lineage, the number of genetic variations inherited from Nanyang cattle is much higher than that inherited from Piedmontese cattle. The nucleotide diversity of Pinan cattle is lower than that of Chinese indicine and other native Chinese cattle breeds, but about twice that of European cattle breeds such as Angus, Hereford, and Piedmontese. The higher genomic diversity of Pinan cattle may be due to the influence of female parent Nanyang cattle. Because Nanyang cattle is a hybrid between *Bos taurus* and *Bos indicus*, it has high genomic diversity. In addition, cattle breeds with higher genetic diversity tend to have lower LD decay, such as Qinchuan cattle. Interestingly, the LD decay level of Pinan cattle was higher than that of Nanyang cattle and Piedmontese, which may be due to the greater selection intensity experienced by the Pinan herd than Nanyang cattle and Piedmontese.

In addition, we found important selective scanning markers in Pinan cattle. Over the past 30 years, Pinan cattle have been used for intensive beef breeding, leading to genetic improvement of production traits, especially for their early onset of puberty. Several genes (*REV3L*, *NT5E*, *CDX2*, *KDM6B*, *ADAMTS9*) in the Pinan cattle genome show selectivity for embryo development. To better understand the selection pressure, we explored the biological function of these genes. *REV3L* is involved in the DNA repair process, and it was upregulated in cattle embryos derived from depleted cells [54]. Loss of *REV3L* resulted in chromosome instability and embryogenesis failure in mice [25]. Genome-wide association study has shown that *REV3L* is associated with embryonic development in Nellore cattle [55]. *NT5E* produces a protein that encodes an enzyme that converts adenosine monophosphate into adenosine [28]. The study has shown that the *NT5E* gene affects the cleavage rate of cattle [56]. In addition, two non-synonymous SNP in *NT5E* can be useful for improving Inosine 5’-monophosphate which contributes to the umami taste in beef in Japanese Black beef [57]. *CDX2* is a transcription factor that is localized in the nucleus of the trophectoderm [58]. Studies have shown that *CDX2* is essential for early development and gene expression involved in the differentiation of inner cell mass and trophectoderm lineages in cattle embryos [30, 59]. *KDM6B*, also known as *JMJD3*, is a histone demethylase expressed in bovine cleavage embryos [60]. *KDM6B* is transiently upregulated around embryo developmental stages when the embryo genome is activated [61]. Another study has shown that H3K27me3 demethylase activity mediated by *KDM6B* is required for normal bovine embryo development and reprogramming gene expression during the embryonic genome [41]. *ADAMTS9* is widely expressed during mouse embryo development [49]. Other studies have shown that *ADAMTS9* is necessary for ovarian development and may promote the rupture for follicular rupture in zebrafish [62, 63]. A study has shown that ADAMTS proteases play a role in remodeling the bovine ovulatory follicle in preparation for ovulation and the formation of the corpus luteum [64]. Interestingly, a GWAS study reported that *ADAMTS9* was associated with chest size in goats [65]. This suggests it may also play an important role in cattle. These genes may be an important reason for the early maturity and faster growth of Pinan cattle than Nanyang cattle.

Native Chinese cattle tend to be more resistant and adaptable than foreign breeds. In a selective sweep of Pinan cattle populations, we identified several immune system-related genes (*BOLA-DQA2*, *BOLA-DQB*, *LSM14A*, and *NAALADL2*). *BOLA-DQA2* and *BOLA-DQB* both belong to the Bovine Leukocyte Antigen (BOLA) class II genes which are involved in the immune response. For example, *BOLA-DQA2* plays an important role in resistance to mastitis in dairy cows [52]. *LSM14A* plays a critical role in antiviral immune responses [33, 66]. *NAALADL2* is reported to be responsible for immune homeostasis [67]. They may have a similar effect in cattle but there are currently few studies on that. In addition, we found a positive selection gene (*ANTXR2*) associated with climate adaptation [21]. These positive selection genes are likely to improve the survival of Pinan cattle.

In addition, we found several genes (*CYP4A11*, *RPL26*, and *MYH10*) related to growth traits. *CYP4A11* is involved in fatty acid metabolism, blood pressure regulation, and kidney tubule absorption of ions [68]. The study has shown that *CYP4A11* affects growth and fat deposition in cattle [17]. *RPL26*, a ribosomal protein gene, is reported to be related to residual feed intake in Holstein cows [69]. GWAS analysis has shown that one missense variant in *RPL26* is associated with average daily gain. There is a report shows that *MYH10* is strongly associated with milk production and body conformation traits of dairy cattle [70]. GWAS study also shows that *MYH10* is a candidate gene for yearling weight in Chinese Simmental beef cattle. In addition, two other positive selection genes *ODF4* and *ARHGEF15* have been reported to be involved in the milk yield of dairy goats. This suggests they may have a similar role in cattle.

## Conclusions

In this study, genomic variation of Pinan cattle was studied comprehensively using WGS data. The study on the population structure and genomic diversity of Pinan cattle will provide the basis for the genetic evaluation of this breed and point out the direction for the development of reasonable breeding strategies for future breeding and improvement. In addition, we have identified a series of candidate genes that may have important effects on survival, growth, early maturity, and meat quality traits of this variety. These results will provide a basis for further genomic studies of beef cattle and provide a reference for genomic studies of imported crossbred beef cattle in China or other parts of the world.

## Methods

### Samples and sequencing

We collected blood samples from 30 Pinan cattle in Xinye County, Henan Province using EDTA-K2 anticoagulant tubes. After dry ice storage, genomic DNA was extracted in the laboratory using the standard phenol–chloroform method [71]. The integrity of genomic DNA was verified by agarose gel electrophoresis. And we used Nanodrop to measure the purity of DNA. DNA samples containing more than 1.5 µg were used to build libraries. A paired-end library with an average insert length of 500 bp and an average read length of 150 bp was constructed for each individual. Sequencing was performed using Illumina Hiseq 2000 platform at the Novogene Bioinformatics, Beijing, China.

To better compare the genetic diversity of Pinan cattle with other cattle breeds in the world, we collected additional samples based on the five "core" groups proposed by Chen et al., [2] as well as the data of 30 Jiaxian cattle and 30 Qinchuan cattle of local breeds in China. The samples include European varieties (Angus, Hereford, Red Angus, Gelbvieh, Limousin, Simmental, Piedmontese, Korean breed (Hanwoo), Chinese breed (Tibetan, Qinchuan, Leiqiong, Guangfeng, Ji’an Wannan and India-Pakistan cattle breeds ((Tharparkar, Sahiwal, Hariana, Nelore, and Gir) (Table S2). A total of 199 samples were used in this study.

### Reads mapping, SNP calling, and annotation

After obtaining the fastq file of the raw data, Trimmomatic software (v0.36) was used to trim sequence reads with the parameters: “LEADING:20, TRAILING:20, SLIDINGWINDWOE: 3:15, AVGQUAL:20, MINLEN:35, TOPHRED33”. The clean reads were aligned to the *Bos taurus* reference genome ARS-UCD1.2 by the “sentieon bwa mem” module with default parameters from sentieon software (versionV202010) [11]. After that “sentieon driver” module was used to filter duplicate reads and used to perform Base Quality Score Recalibration with default parameters. Single nucleotide polymorphisms (SNPs) were detected by the Genome Analysis Toolkit (GATK, version 4.1.8.1) [72]. The raw SNPs were called using the “SelectVariants” module of GATK. After SNPs were called, the “VariantFiltration” module was used to filter SNPs with the parameters “QualByDepth (QD) < 2.0, Quality (QUAL) < 30.0, StrandOddsRatio (SOR) > 3.0, FisherStrand (FS) > 60.0, RMSMappingQuality (MQ) < 40.0, MappingQualityRankSumTest (MQRankSum) < -12.5, and (ReadPosRankSumTest) ReadPosRankSum < -8.0”. Finally, a total of 53,174,296 biallelic SNPs were obtained using bcftools (version 1.8) with the parameters: F_MISSING < 0.1 & MAC > 2. After that, SNPs were filtered using VCFtools v0.1.16 with the parameters: –maf 0.05 and –max-missing 0.9. Based on the *Bos taurus* reference genome annotation file(https://ftp.ncbi.nlm.nih.gov/genomes/all/GCF/002/263/795/GCF_002263795.1_ARS-UCD1.2/GCF_002263795.1_ARS-UCD1.2_genomic.gff), Annovar [73] software was used to annotate the functions of each SNP.

### Population genetic analysis

PLINK (version 1.9) [74] software was used to remove those sites with high LD with the parameter (–indep-pair-wise 50 5 0.2), and the remaining SNP sites were used for performing ADMIXTURE analysis. PCA was performed using the SmartPCA program in the Eigensoft V5.0 package [75]. ADMIXTURE v1.3 [76] was used for population structure analysis, the kinship set is from 2 to 8. The matrix of pairwise genetic distances supplied by plink was used for constructing an unrooted evolutionary tree. The visualization of evolutionary was done by MEGA7 v7.0.26 [77] and FigTree v1.4.4 (http://tree.bio.ed.ac.uk/software/figtree/).

### Selective sweep identification

In order to detect selective signals in the Pinan cattle, we used different strategies to scan the genomes of Pinan cattle. The nucleotide diversity (θπ) and the composite likelihood ratio (CLR) [78] method were used in the Pinan cattle population. First, the SNP loci with allele frequency less than 0.05 were removed. After that, we used VCFtools v0.1.16 [79] to estimate the nucleotide diversity by a sliding window approach in which the windows were 50 kb and the step size is 20 kb. The SweepFinder2 [80] was used for calculating the CLR with a grid size of 50 kb. In addition, fixation index (F_ST_) and cross-population extended haplotype homozygosity (XP-EHH) were used for comparing the genomic difference between Pinan and Piedmontese cattle. We used VCFtools to analyze F_ST_ with a 50 kb window and a 20 kb step. Within each population, haplotype phasing was performed using BEAGLE 5.0 [81], and selscan V1.1 [82] was used to calculate the XP-EHH statistics for each population. For XP-EHH selection scans, our test statistics are the average of standardized XP-EHH scores for each 50 kb region.

### DCMS estimation

In this study, we combined four statistics including θπ, CLR, F_ST_, and XPEHH into a single DCMS framework as described in Ma et al. [13]. The analysis steps were carried out by referring to other studies [10, 83–85]. The DCMS statistic was calculated for each 50 kb window using the R MINOTAUR package [86]. The genome-wide P-value was calculated by the *stat_to_pvalue* function of the R MINOTAUR package, and appropriate unilateral tests were performed, i.e., a left tailed test for the θπ and CLR (two.tailed = FALSE, right.tailed = FALSE) and a right tailed test for the F_ST_ and XPEHH (two.tailed = FALSE, right.tailed = TRUE). Then, the n × n correlation matrix between the statistics is calculated using the *covNAMcd* function (alpha = 0.75, nsamp = 50,000) of the R rrcovNA package [87]. The matrix was used as the input of the *DCMS* function of the R MINOTAUR package to calculate the DCMS value of the whole genome. After calculating the DCMS value, these DCMS values were transformed to a normal distribution with the robust linear model using the *rlm* function (dcms ~ 1) of the R MASS package [88]. The output of the fitting model is the input of the *pnorm* R function to calculate the p-value of DCMS statistics. To control for multiple test false discovery rate (FDR) in the rejected null hypothesis, the *p.adjust* R function (p = dcms_pvalue, method = ”BH”, n = length(dcms_pvalue)) was adopted to convert the p-value of DCMS into the corresponding q-value according to Benjamini and Hochberg methods.

### Candidate gene annotation

The region with DCMS q-value lower than 0.05 was regarded as the statistically significant region, and the genes contained in the significant region were annotated through the reference genome annotation file based on the ARS-UCD 1.2 reference genome. The function of these candidate genes was further determined through a literature review.

## Supplementary Information

**Additional file 1:** **Figure S1.** Phylogenetic tree constructed by neighbor-joining method from Pinan cattle and some other breeds.

**Additional file 2:** **Table S1.** Summary of sequencing data. **Table S2.** List of additional cattle samples for analysis of genetic background in Pinan cattle. **Table S3.** Distribution of SNPs identified in cattle breeds within various genomic regions annotated by ANNOVAR. Only the breeds with a sample size no less than 5 were calculated in the table. **Table S4.** CV error corresponding to different K values. Table S5. 92 selective signal candidate regions identified by DCMS.

## Data Availability

Sequences are available from GenBank with the Bioproject accession number PRJNA698276 (https://www.ncbi.nlm.nih.gov/bioproject/PRJNA698276).

## Abbreviations

SNP: Single nucleotide polymorphism
WGS: Whole-genome sequencing
LD: Linkage disequilibrium
PCA: Principal component analysis
NJ: Neighbor-joining
θπ: Nucleotide diversity
CLR: Composite likelihood ratio
FST: Fixation index
XP-EHH: Cross-population extended haplotype homozygosity
GO: Gene Ontology
KEGG: Kyoto Encyclopedia of Genes and Genomes
