# Supplementary material for: Assessing genomic diversity and signatures of selection in Pinan cattle using whole-genome sequencing data
Source: BMC Genomics. 2022 Jun 21;23:460. doi: 10.1186/s12864-022-08645-y (PMC9215082; doi:10.1186/s12864-022-08645-y)
Supplement: Supplementary file 1 — Additional file 1: Figure S1. Phylogenetic tree constructed by neighbor-joining method from Pinan cattle and some other breeds. [file 12864_2022_8645_MOESM1_ESM.pdf]

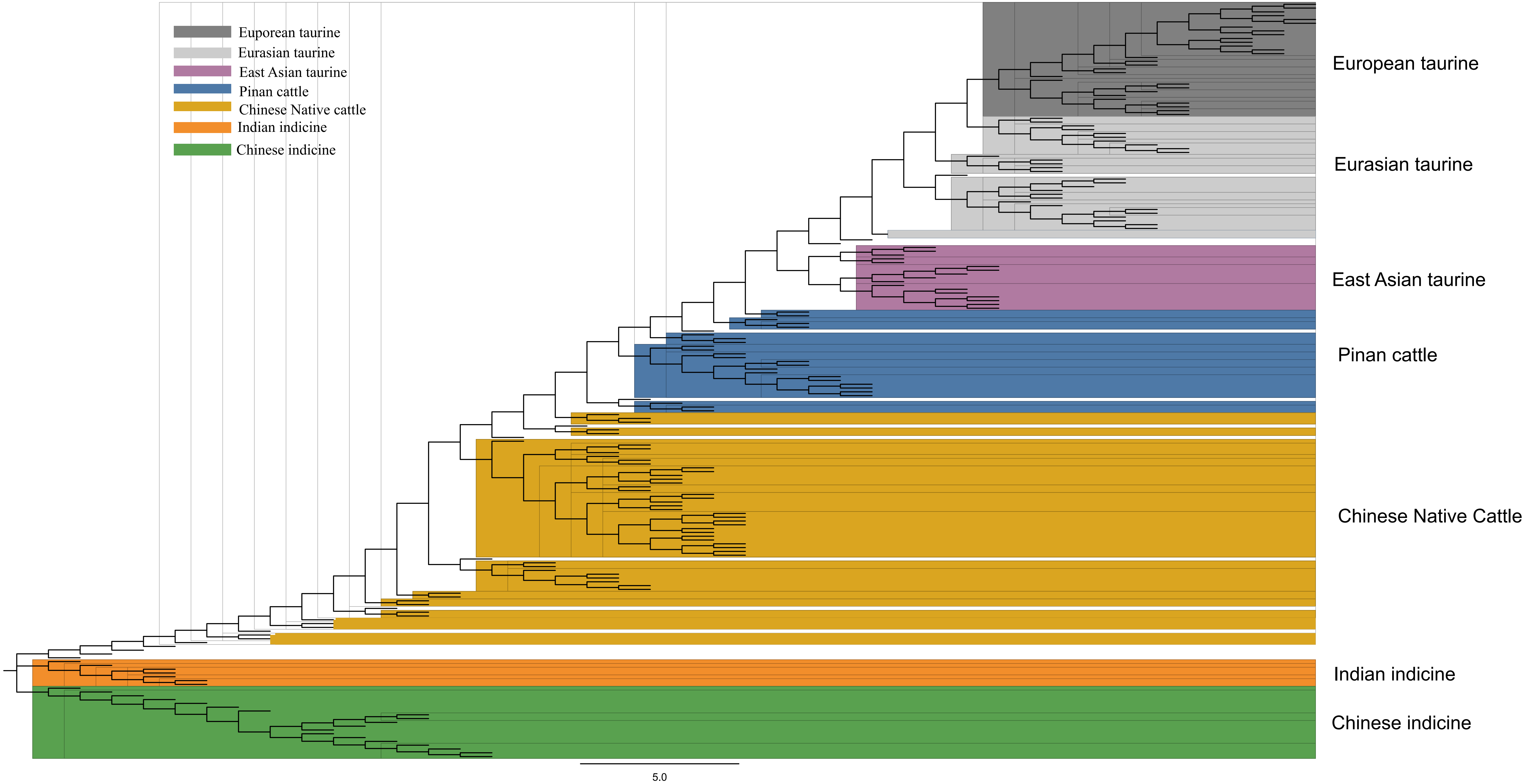

Figure S1. Phylogenetic tree constructed by neighbor-joining method from Pinan cattle and some other breeds.
